# A liquid biomarker signature of inflammatory proteins accurately predicts early pancreatic cancer progression during FOLFIRINOX chemotherapy

**DOI:** 10.1016/j.neo.2024.100975

**Published:** 2024-02-09

**Authors:** Casper W.F. van Eijck, Sergio Sabroso-Lasa, Gaby J. Strijk, Dana A.M. Mustafa, Amine Fellah, Bas Groot Koerkamp, Núria Malats, Casper H.J. van Eijck

**Affiliations:** a Erasmus MC Cancer Institute, Erasmus University Medical Center, Rotterdam, The Netherlands; b Genetic and Molecular Epidemiology Group, Spanish National Cancer Research Center, Madrid, Spain; c Centro de Investigación Biomédica en Red-Cáncer (CIBERONC), Madrid, Spain; d Department of Clinical Bioinformatics, Erasmus University Medical Center, Rotterdam, The Netherlands; e Department of Surgery, Erasmus University Medical Center, Rotterdam, The Netherlands

**Keywords:** Biomarkers, Gene Expression Profiling, folfirinox, Pancreatic Neoplasms, Precision Medicine, Protein Array Analysis

## Abstract

**Background:**

Pancreatic ductal adenocarcinoma (PDAC) is often treated with FOLFIRINOX, a chemotherapy associated with high toxicity rates and variable efficacy. Therefore, it is crucial to identify patients at risk of early progression during treatment. This study aims to explore the potential of a multi-omics biomarker for predicting early PDAC progression by employing an in-depth mathematical modeling approach.

**Methods:**

Blood samples were collected from 58 PDAC patients undergoing FOLFIRINOX before and after the first cycle. These samples underwent gene (GEP) and inflammatory protein expression profiling (IPEP). We explored the predictive potential of exclusively IPEP through Stepwise (Backward) Multivariate Logistic Regression modeling. Additionally, we integrated GEP and IPEP using Bayesian Kernel Regression modeling, aiming to enhance predictive performance. Ultimately, the FOLFIRINOX IPEP (FFX-IPEP) signature was developed.

**Results:**

Our findings revealed that proteins exhibited superior predictive accuracy than genes. Consequently, the FFX-IPEP signature consisted of six proteins: AMN, BANK1, IL1RL2, ITGB6, MYO9B, and PRSS8. The signature effectively identified patients transitioning from disease control to progression early during FOLFIRINOX, achieving remarkable predictive accuracy with an AUC of 0.89 in an independent test set. Importantly, the FFX-IPEP signature outperformed the conventional CA19-9 tumor marker.

**Conclusions:**

Our six-protein FFX-IPEP signature holds solid potential as a liquid biomarker for the early prediction of PDAC progression during toxic FOLFIRINOX chemotherapy. Further validation in an external cohort is crucial to confirm the utility of the FFX-IPEP signature. Future studies should expand to predict progression under different chemotherapies to enhance the guidance of personalized treatment selection in PDAC.

## Highlights

• The FOLFIRINOX inflammatory protein expression profiling (FFX-IPEP) signature effectively identified patients who progressed early during FOLFIRINOX.
• The FFX-IPEP liquid biomarker, comprising AMN, BANK1, IL1RL2, ITGB6, MYO9B, and PRSS8, surpasses CA19-9 in predicting PDAC progression under FOLFIRINOX.
• The FFX-IPEP showed predictive accuracy across all disease stages, removing the need to factor in disease stage for FOLFIRINOX response assessment.
• Multi-omics mathematical modeling underscored the predictive accuracy of proteins over genes for PDAC progression under FOLFIRINOX.

## Introduction

The five-year overall survival (OS) rate of pancreatic ductal adenocarcinoma (PDAC) remains less than 10 % for all stages [1]. In Europe, PDAC is the third leading cause of cancer-related death despite being the seventh most common cancer [2]. Approximately 80 % of PDAC patients present with unresectable advanced-stage disease [3] due to, amongst others, early and progressive local and distant disease spread [1] and the lack of reliable biomarkers for early detection [4]. Even those patients who undergo successful surgery experience disease recurrence in approximately 70 % of cases [5], with a marginal increase in the five-year OS to less than 10 % [6]. Other emerging therapies have had limited success in improving survival rates in recent years [7], primarily due to the heterogeneous and complex immunosuppressive phenotype of PDAC [8].

Nonetheless, the multi-agent chemotherapy consisting of 5-FU, folinic acid, irinotecan, and oxaliplatin (FOLFIRINOX) has become a dominant treatment option for all stages of PDAC, exhibiting superior efficacy and cost-effectiveness compared to gemcitabine-based treatments [9]. Randomized controlled trials demonstrated prolonged OS with FOLFIRINOX, both as first-line treatment for metastatic PDAC (11 months vs. 7 months) [10] and as adjuvant treatment for resectable PDAC (54 months vs. 36 months)[11]. Moreover, meta-analyses have reported improved OS with FOLFIRINOX as first-line treatment for locally advanced pancreatic cancer (LAPC) (24 months vs. 6-13 months) [12] as well as neoadjuvant treatment in borderline resectable PDAC [[13], [14]].

Despite its promising efficacy, the use of FOLFIRINOX is often limited by the need for dose reductions due to high toxicity rates [10,12,15,16]. Additionally, roughly 25 % of patients still experience disease progression under treatment [10,16]. The dual risk of ineffectiveness and toxicity treatment tempers enthusiasm among physicians and patients when considering FOLFIRINOX as a treatment option. Early identification of patients likely to progress under FOLFIRINOX is crucial to overcome these concerns and optimize treatment decisions, especially given the typically poor survival rates in advanced PDAC [17]. However, radiological assessment of FOLFIRINOX response is conventionally scheduled after completing four cycles, as earlier radiological responses are typically not observed. Furthermore, carbohydrate antigen 19-9 (CA19-9), a Food and Drug Administration (FDA) approved biomarker for the routine management of PDAC [18], cannot predict FOLFIRINOX response early on [19], only after multiple cycles [20]. In addition, about 7 % of the population cannot express CA19-9, rendering the biomarker futile in these cases [21].

In a prior study, we demonstrated the potential of a multigene gene expression profiling (GEP) score to predict unresponsiveness to FOLFIRINOX in PDAC patients [19]. Nevertheless, proteins represent an appealing biomarker alternative due to their cost-effectiveness, broad accessibility, and smooth integration into routine clinical practice, resulting in fast reporting times [22]. Subsequently, we delved into the proteome profiles of PDAC patients and highlighted the promise of standalone protein biomarkers [23]. However, in that study, our primary focus was to understand the immunological effects of FOLFIRINOX in guiding more effective combination therapies rather than modeling the collective predictive potential of multiple proteins. In this follow-up study, we used comprehensive mathematical modeling to evaluate the efficacy of a liquid biomarker signature featuring multiple inflammatory proteins for predicting early PDAC progression during FOLFIRINOX chemotherapy. The developed FOLFIRINOX inflammatory protein expression profiling (FFX-IPEP) signature demonstrated remarkable predictive capability. Furthermore, our findings indicated that models integrating GEP and IPEP did not enhance predictive capability.

## Materials and Methods

### Patient population and clinical procedures

This study included patients with histologically confirmed PDAC, aged 18 or older, who had not undergone prior chemotherapy for PDAC. Patients with LAPC and metastatic PDAC originated from the prospective iKnowIT trial (Dutch trial register NL7522), and patients with (borderline) resectable PDAC originated from the randomized clinical PREOPANC-2 trial (Dutch trial register NL7094). All patients received at least four cycles of FOLFIRINOX between February 2018 and February 2021. To reduce FOLFIRINOX-induced neutropenia, patients were prophylactically treated with the long-acting granulocyte-colony stimulating factor (G-CSF) lipegfilgrastim (Lonquex®, Teva Ltd, Petach Tikva, Israel), 24 hours after each cycle [24].

**Fig. 1:**
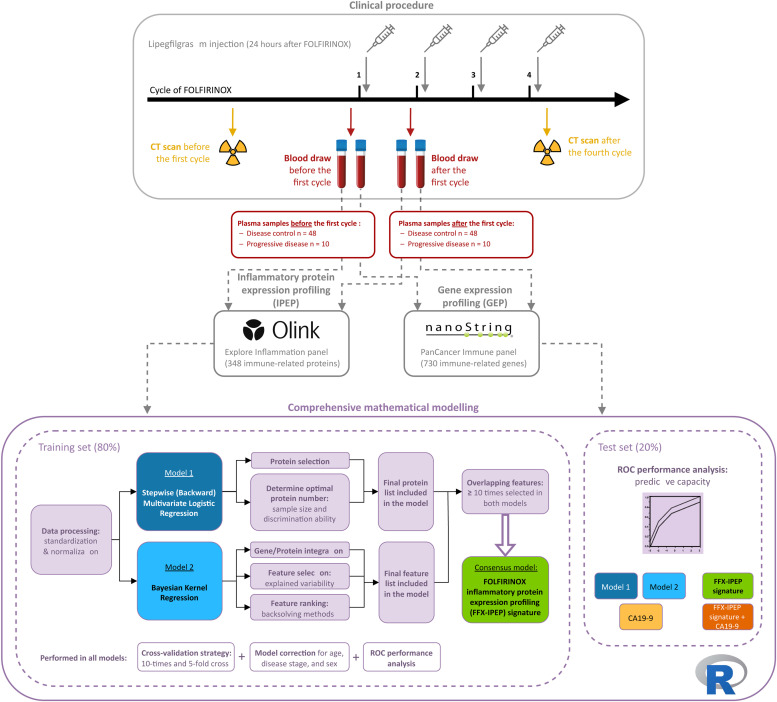
Schematic overview of the methodological steps. The squircles illustrate the methodological steps of the study: (1) clinical procedure, (2) IPEP and GEP profiling, and (3) comprehensive mathematical modeling. Abbreviations: CA19-9, Carbohydrate Antigen 19-9; CT, Computed Tomography; FFX, FOLFIRINOX, GEP, Gene Expression Profiling; IPEP, Inflammatory Protein Expression Profiling; ROC, Receiver Operating Characteristic.

A schematic overview of the methodological steps can be found in Fig. 1. Blood samples were drawn from each patient at two time points in duplicate, resulting in four blood samples per patient for both GEP and IPEP. The first sample was taken at baseline (within one week before the first cycle), and the second was taken approximately two weeks after the first FOLFIRINOX cycle (within one week before the second cycle). In addition, standard laboratory parameters, including CA19-9, were measured. Radiological response to FOLFIRINOX was assessed by comparing computed tomography (CT) scans taken at baseline and after the fourth FOLFIRINOX cycle, following the Response Evaluation Criteria in Solid Tumors (RECIST) 1.1. Radiologists responsible for assessing the response were blinded to the omics results. Patients were divided into two groups based on radiological response: those who showed stable disease, partial response, or complete response were defined as having “disease control”, while patients showing disease progression were defined as having “progressive disease”.

### Gene expression profiling (GEP) and inflammatory protein expression profiling (IPEP)

The blood samples were collected in Tempus tubes (Applied Biosystems, Foster City, CA, USA) and Vacutainer plasma separator tubes (Becton Dickinson, Franklin Lakes, NJ, USA) and for GEP end IPEP, respectively, and cryopreserved at -80°C until further analysis. Details on targeted multiplex GEP using the nCounter FLEX system of NanoString Technologies (Seattle, WA, USA) and plasma IPEP using the Olink Proteomics platform (Uppsala, Sweden) are available in **the supplementary methods** and have been previously described [19,23].

### Statistical analysis

We employed a comprehensive mathematical modeling approach to predict PDAC progression under FOLFIRINOX. In cases where missing values occurred, k-nearest neighbors (KNN) imputation models (k = 20) were employed using information from the remaining non-null values. The expression datasets were divided into an 80 % training set and a 20 % test set for model development. The performance of the models was evaluated using Receiver Operating Characteristic (ROC) analysis and the area under these curves (AUC). This assessment involved a 10-times 5-fold cross-validation strategy on the training set, with balanced partitions for the response to FOLFIRINOX. Importantly, we considered the time of blood draw evaluation (before and after the first cycle) as an interaction factor. Due to its superior predictive performance, this approach was preferred over delta values (the difference between feature values at baseline and after the first. All models were adjusted for age, sex, and disease stage.

Three models were constructed: one solely based on IPEP, another integrating IPEP and GEP, and the consensus one that merged both. Several alternative machine-learning models for feature selection were considered, including Random Forests, Support Vector Machines, and personalized regression models like LASSO, Ridge, and ElasticNet. Nevertheless, the models mentioned below consistently demonstrated superior predictive performance.

In the first model, we evaluated the predictive potential of IPEP data through a Stepwise (Backward) Multivariate Logistic Regression model. This approach generated a final protein list in each cross-validation iteration, ranked according to their selection frequency across 50 iterations. Subsequently, a sample size and discrimination ability assessment determined the number of proteins that should be selected from this list and evaluated in an independent test dataset.

In the second model, we introduced gene expression data used in our previously developed FOLFIRINOX delta gene expression profiling (FFX-ΔGEP) score [19] to explore its potential to enhance the model's predictive capability solely reliant on proteins. Given the challenge of having more variables than samples (p >> n), we implemented Bayesian Kernel Regression models [25] within the same train and test partitions employed in the previous model. This method allowed us to create similarity matrices or kernels, effectively reducing dimensionality to match the number of individuals in the dataset. For all 50 iterations, we determined the variability explained by the features within the model. Specifically, the response to FOLFIRINOX was expressed by the following equation: $y\;=\;X\beta \;+\;Zu_{\mathrm{prot}\mathrm{ein}}+\;Zu_{\mathrm{gene}}+\;\varepsilon$, where y is the response to FOLFIRINOX (disease control or progressive disease); β is the clinical effects vector; X is the incidence matrix related to clinical variables; Z is an identity matrix; $u_{\mathrm{protein}}$ and $u_{\mathrm{gene}}$ are vectors corresponding to prognostic scores for each individual and data layer; and ɛ is the error vector. Both similarity matrices were constructed as the (co)variance matrix using the already scaled and centered incidence matrix. The Bayesian Generalized Linear Regression (BGLR) R package [26] was used to estimate the unknown parameters, comprising the variance components (one for each data layer and the residual variance) and prognostic scores for each data layer. In addition, using various back-solving techniques, we also determined and ranked the features that exerted the greatest impact on this variability [19,27,28].

The third consensus model, defined as the FOLFIRINOX IPEP (FFX-IPEP) signature, comprised the proteins from the first model that were well-ranked in the overlapping features selected at least ten times in both models. Feature collinearity was evaluated through the variance inflation factor (VIF), with a VIF ≥ 5 indicating collinearity per established practice [29]. Additionally, we analyzed the predictive capacity of normalized CA19-9 levels, considering its potential use in predicting FOLFIRINOX response [20]. We directly compared CA19-9′s predictive capacity with other models and investigated the impact of its inclusion in the consensus model on predictive performance. The blood draw time was considered an interaction factor rather than calculating delta values, aligning with the methodology for GEP and IPEP. Furthermore, CA19-9 levels were measured concurrently at the same two time points as GEP and IPEP. Patients with levels < 35 U/mL at either time point were excluded from this analysis, as they were considered not to express CA19-9.

Statistical analyses and visualizations for all datasets were conducted using R Statistical Software (v4.1.2), and p values < 0.05 were considered statistically significant. If applicable, p values were adjusted for multiple hypothesis testing using the Benjamini-Hochberg correction to calculate the false discovery rate (P.adj). To assess the normality of clinical characteristics, we employed the Shapiro-Wilk test. Since not all characteristics exhibited a normal distribution, continuous variables were presented as median values (minimum, maximum), while categorical variables were expressed as total numbers with relative frequencies. Statistical testing of clinical characteristics between treatment response subgroups was performed using the Mann-Whitney-Wilcoxon test for continuous variables and Pearson's chi-square and Fisher tests for categorical variables.

## Results

### Patient characteristics

Two peripheral blood samples were collected from 58 PDAC patients before and after the first cycle of FOLFIRINOX chemotherapy, resulting in 116 samples. Due to the inherent nature of our analysis after only the first cycle, the treatment response subgroups were unbalanced, with 48 patients displaying disease control and 10 patients showing progressive disease. Importantly, the progressive disease rate was similar across PDAC stages: 10 % in (borderline) resectable, 25 % in LAPC, and 20 % in metastatic patients. There were no significant differences in clinical characteristics between disease control and progressive disease patients, except for the number of FOLFIRINOX cycles, which was lower in patients with progressive disease due to treatment discontinuation upon disease progression (Table 1). All samples underwent gene expression profiling (GEP) and inflammatory protein expression profiling (IPEP) and met the manufacturer's quality standards.

**Table 1:** Clinical characteristics of the PDAC patient cohort (n = 58).

**Treatment response**	**Disease control (n = 48)**	**Progressive disease (n = 10)**	**P value**	**Total cohort (n = 58)**
**RECIST1.1 after four cycles, n (%)**				
Partial response	12 (25)	0 (0)	–	12 (21)
Stable disease	36 (75)	0 (0)		36 (62)
Progressive disease	0 (0)	10 (100)		10 (17)
**Disease stage, n (%)**				
(Borderline) resectable disease	18 (38)	2 (20)	0.51	20 (34)
Locally advanced (LAPC)	18 (38)	6 (60)		24 (41)
Metastatic disease	12 (25)	2 (20)		14 (24)
**Median age [min, max]**	65 [48, 78]	62 [47, 68]	0.19	64 [47, 78]
**Median BMI (kg/m2) [min, max]**	25 [16, 36]	25 [19, 36]	0.91	25 [16, 36]
**Sex, n (%)**				
Female	23 (48)	4 (40)	0.74	27 (47)
Male	25 (52)	6 (60)		31 (53)
**Alcohol use, n (%)**				
Current	23 (48)	3 (30)	0.57	26 (45)
Former	7 (15)	2 (20)		9 (16)
Never	18 (38)	5 (50)		23 (40)
**Smoking status, n (%)**				
Current	11 (23)	2 (20)	1.0	13 (22)
Former	18 (38)	4 (40)		22 (38)
Never	19 (40)	4 (40)		23 (40)
**Diabetes Mellitus, n (%)**				
No	38 (79)	7 (70)	0.68	45 (78)
Yes	10 (21)	3 (30)		13 (22)
**History of malignancy, n (%)**				
No	42 (88)	8 (80)	0.62	50 (86)
Yes	6 (12)	2 (20)		8 (14)
**History of pancreatitis, n (%)**				
No	46 (96)	9 (90)	0.44	55 (95)
Yes	2 (4)	1 (10)		3 (5)
**CA19-9 (U/mL), median [min, max]**				
Before first cycle	294 [0, 26500]	418 [4, 83300]	0.41	294 [0, 83300]
After first cycle	207 [0, 35700]	380 [5, 85200]	0.28	207 [0, 85200]
Absolute change	5 [-5860, 9260]	13 [-3670, 4070]	0.46	9 [-5860, 9260]
Non-expressors, n (%)	8 (17)	0 (0)		8 (14)
**CEA (ng/L), median [min, max]**				
Before first cycle	4.1 [0.8, 119]	3.8 [0.7, 226]	0.61	4.1 [0.7, 226]
After first cycle	5.4 [0.9, 139]	6.0 [1.2, 241]	0.36	5.9 [0.9, 241]
Absolute change	0.4 [-13, 20]	0.7 [-2.8, 15]	0.18	0.5 [-13, 20]
Missing, n (%)	10 (21)	1 (10)		11 (19)
**NLR, before first cycle, median [min, max]**				
Before first cycle	3.1 [1.1, 20]	3.2 [1.7, 6.9]	0.45	3.3 [1.2, 20]
After first cycle	4.9 [1.6, 20]	3.2 [0.9, 7.6]	0.24	4.9 [0.9, 20]
Absolute change	1.7 [-13, 15]	0.8 [-2.0, 4.3]	0.31	1.4 [-13, 15]
Missing, n (%)	6 (13)	3 (30)		9 (16)
**SII, before first cycle, median [min, max]**				
Before first cycle	870 [264, 7250]	1010 [468, 2380]	0.27	881 [264, 7250]
After first cycle	1110 [366, 2870]	962 [202, 2710]	0.63	1070 [202, 2870]
Absolute change	193 [-6180, 1750]	196 [-1530, 1690]	0.94	194 [-6180, 1750]
Missing, n (%)	6 (13)	3 (30)		9 (16)
**Median FOLFIRINOX cycles [min, max]**	8 [3, 10]	4 [1, 8]	**< 0.001**	8 [1, 10]

Abbreviations: BMI, Body Mass Index; CEA, Carcinoembryonic Antigen; NLR, Neutrophil-to-Lymphocyte Ratio; RECIST1.1, Response Evaluation Criteria In Solid Tumors 1.1; SII, Systemic Immune-Inflammatory Index.

### Modeling PDAC progression under FOLFIRINOX using protein data

**Fig. 2:**
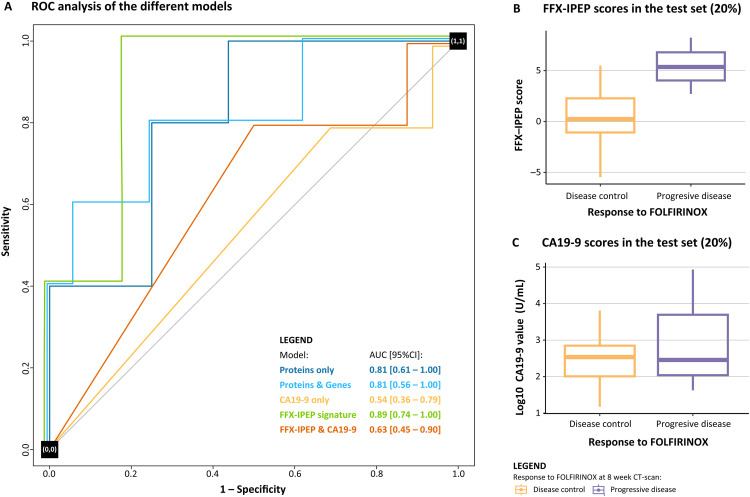
**Performance analysis of constructed models. (A)** ROCs illustrating the predictive performance of various models for early PDAC progression under FOLFIRINOX in the independent test set (20 %). Each colored line represents a different model, with the y-axis indicating sensitivity and the x-axis displaying 1–specificity. Notably, the FFX-IPEP signature demonstrated the highest AUC of 0.89 [95 % CI: 0.74 – 1.00]. **(B)** Boxplot illustrating the FFX-IPEP scores (y-axis) assigned to individual patients in the test set (20 %), stratified by treatment response (disease control and progressive disease). **(C)** Boxplot illustrating the log10 CA19-9 values in U/mL (y-axis) of individual patients in the test set (20 %), stratified by treatment response (disease control and progressive disease). Abbreviations: AUC, Area Under the Curve; CA19-9, Carbohydrate Antigen 19-9; FFX, FOLFIRINOX; IPEP, Inflammatory Protein Expression Profiling; ROC, Receiver Operating Characteristic.

The first model exclusively contained IPEP data and was constructed through Stepwise (Backward) Multivariate Logistic Regression modeling. **Supplementary Table S1** presents the frequency of protein selection following 50 iterations. To identify the optimal number of proteins and prevent overfitting, we conducted an incremental AUC analysis by adding one selected protein in each iteration. This analysis revealed a risk of overfitting when more than eight proteins were selected (**Supplementary Figure S1**). Consequently, the top eight ranked proteins were selected, which included AMN, BACH1, BANK1, HEXIM1, IL1RL2, ITGB6, MYO9B, and PRSS8 (Table 2, **Supplementary Figure S2**). These proteins were combined into a Multivariate Logistic Regression model to predict PDAC progression under FOLFIRINOX. The ROC performance analysis yielded an AUC [95 %CI] of 0.97 [0.93 – 1.00] for the training set and 0.81 [0.61 – 1.00] for the independent test set, both assessed separately from the cross-validation (Fig. 2A).

**Table 2:** Literature summary of the characteristics of the proteins included in the models.

**Protein**	**Full name**	**Aliases**	**Model**	**Blood upregulation in disease [**30]	**Tissue and immune cell expression [**30]	**Molecular and biological function**	**Function oncology**	**Function chemosensitivity**
**AMN**	Vitamin Transport Protein Amnionless	Amnionless, IGS2, PRO1028	Protein only, FFX-IPEP	Myeloma.	Cytoplasmic expression in several tissues. No expression on immune cells.	Transmembrane protein crucial for facilitating vitamin B12 absorption and transport [31].	Pro-tumoral role in colorectal cancer. Downregulation is associated with carcinogenesis and poor survival [32].	–
**BACH1**	BTB domain and CNC homolog 1	BTBD24	Protein only	CLL, AML, myeloma, DLBCL, prostate cancer, breast cancer.	Nuclear and cytoplasmic expression in several tissues. Expression on various immune cells, especially on neutrophils.	Transcription factor serving facilitating DNA binding. Regulates ROS production, cell cycle, hematopoiesis, immunity, and involved in cardiovascular diseases [33].	Pro-tumoral role in PDAC. Activates pro-metastatic genes, resulting in cell migration and invasion [34]. High IHC expression associated with poor prognosis [35].	Promotes resistance to gemcitabine in PDAC [36]
**BANK1**	B Cell Scaffold Protein with Ankyrin Repeats 1	BANK, FLJ20706	Protein only, protein/gene, FFX-IPEP signature	CLL, breast cancer, prostate cancer, myeloma.	Cytoplasmic expression in lymphoid tissue. Expression on various immune cells, especially memory naive B cells.	B cell signaling protein that activates and modulates BCR, CD40, and TLR signaling pathways [33].	Anti-tumoral role in B cell lymphoma, inhibiting proliferation [37]. Downregulation is a valuable biomarker for colorectal cancer [38].	–
**CCL17**	C-C Motif Chemokine Ligand 17	ABCD-2, SCYA17, TARC	Protein/gene	Lung cancer, colorectal cancer, prostate cancer, breast cancer.	Cytoplasmic expression in most tissues and in immune cells.	Cytokine ligand for CCR4 involved in chemotaxis and facilitates the recruitment of T helper 2 and T regulatory cells [39].	Dual role in oncology. Associated with poor survival in squamous cell carcinoma and breast cancer. Associated with better survival in clear cell renal cell carcinoma, lung cancer, and melanoma. Promotes TILs infiltration but attracts Tregs [39].	–
**CCL20**	C-C Motif Chemokine Ligand 20	CKb4, LARC, MIP-3a, SCYA20, ST38	Protein/gene	Myeloma, colorectal cancer, lung cancer.	Expression on various immune cells, especially MAIT cells.	Cytokine ligand for CCR6 involved in chemotaxis. Facilitates the recruitment of T helper 17 cells and Tregs [40].	Pro-tumoral role in oncology. Contributing to the progression of multiple cancers, including PDAC, and promotes immune evasion [40,41].	Mediates chemoresistance to taxane in breast cancer [42], to cisplatin[43] and doxorubicin [44] in ovarian cancer, and FOLFOX in colorectal cancer [45,46].
**FCRL3**	Fc Receptor-Like 3	CD307c, FCRH3, IFGP3, IRTA3, SPAP2a-e	Protein/gene	CLL, DLBCL, myeloma.	Expression on various immune cells, especially naive B cells.	Receptor protein that mediates BCR signaling, plasma B cell maturation, and antibody production [47]. Associated with various autoimmune diseases [48,49].	Anti-tumoral role in oncology. Higher expression associated with an improved prognosis in cervical cancer [50]. Genetic polymorphisms are linked to the risk of head and neck cancer [51].	–
**HEXIM1**	Hexamethylene bisacetamide-inducible protein 1	CLP-1, EDG1, HIS1, MAQ1	Protein only	CLL, AML, colorectal cancer, myeloma, lung cancer.	Ubiquitous nuclear expression. Low immune cell specificity	Transcription factor that regulates RNA elongation and immunity. It is also acts as a tumor suppressor [52].	Anti-tumoral role in oncology. Positive regulator of the p53 tumor suppressor [53]. Inhibition of breast cancer metastasis by regulating cell invasion and angiogenesis [54].	–
**IL1RL2**	Interleukin 1 Receptor-Like 2	36R, IL1R-rp2, IL1RRP2	Protein only, FFX-IPEP signature	Glioma, breast cancer, prostate cancer.	No expression on immune cells.	Receptor for IL36. Involved in immune regulation, inflammatory responses, and tissue remodeling [55].	Pro-tumoral role in colon cancer. Decreased tumoral expression of IL36R is associated with improved outcomes and decreased progression [56,57].	–
**ITGB6**	Integrin Beta-6	–	Protein only, FFX-IPEP signature	Prostate cancer, lung cancer.	Cytoplasmic and membranous expression in various tissues, especially the kidney, skeletal muscle, and tongue. Low immune cell specificity.	Cell surface protein of the integrin family that functions as a host cell receptor for virus entry, playing a vital role in cell adhesion, tissue remodeling, wound healing, and TGF-beta activation [58].	Pro-tumoral role in PDAC. Contributes to disease progression by promoting malignant cellular behavior and is associated with a worse prognosis [59], [60], [61].	Found to hold promise as a standalone biomarker for the early prediction of pancreatic cancer progression during FOLFIRINOX [23].
**MYO9B**	Integrin Beta-6	CELIAC4	Protein only, protein/gene, FFX-IPEP signature	CLL, glioma.	General cytoplasmic expression. No expression on immune cells.	Myosin protein involved in actin-binding, calmodulin-binding, GTPase-activation. Regulates the actin cytoskeleton, cell motility, and intra-cellular transport, with implications in IBD [62].	Pro-tumoral role in various cancers. Promotes disease progression and cancer cell migration in lung and prostate cancer and is associated with poor survival [63,64]. Knockdown suppresses breast cancer cell proliferation, migration, and invasion [65].	Found to hold promise as a standalone biomarker for the early prediction of pancreatic cancer progression during FOLFIRINOX [23].
**PNLIPRP2**	Pancreatic Lipase-Related Protein 2	PLRP2	Protein/gene	–	No expression on tissue or immune cells.	Pancreatic enzyme. Involved in fatty acid turnover, digestion, and signaling [66].	The tumoral role in PDAC is unknown but found to be downregulated in PDAC tissue [67].	–
**PRSS8**	Serine Protease 8	CAP1	Protein only, protein/gene, FFX-IPEP signature	Ovarian cancer, lung cancer.	Cytoplasmic expression in several tissues, especially the salivary gland. No expression on immune cells.	Serine protease enzyme crucial for proteolysis, sodium balance, glucose homeostasis, and the maintenance of epidermal barrier function [68].	Anti-tumoral role in oncology [69] Inhibits breast cancer invasiveness [70], suppresses tumorigenesis in colorectal [71] and hepatocellular cancer [72], and inhibits EMT in human bladder cancer cells [73].	Found in a gene signature to predict chemosensitivity to cisplatin, docetaxel, paclitaxel, and vinblastine in bladder cancer [74]. It also contributes to paclitaxel and cisplatin chemoresistance in ovarian cancer [75].
**TNFAIP8**	Tumor Necrosis Factor Alpha-Induced Protein 8	GG2-1, MDC-3.13, SCC-S2	Protein/gene	Chronic lymphocytic leukemia.	Cytoplasmic expression in lymphoid tissues and the gastrointestinal tract. Low immune cell specificity.	Protein member of the TNFAIP8/TIPE family. Involved in apoptosis regulation, immune homeostasis, cellular processes, proliferation, and cell apoptosis inhibition [76].	Pro-tumoral role in oncology. Associated with poor prognosis of various cancers, affecting cell proliferation, apoptosis, invasion, and metastasis [77,78]. In PDAC, it is highly expressed and closely linked to the EGFR upregulation [77,79].	Promotes chemoresistance to cisplatin and paclitaxel in NSCLC [80,81], to cisplatin and nedaplatin in cervical cancer [82], to platinum, cisplatin, and paclitaxel in ovarian cancer [83,84], and to cisplatin in esophageal cancer [85].

Abbreviations: AML, Acute Myeloid leukemia; BCR: B-Cell Receptor; CCR: C-C Chemokine Receptor; CLL: Chronic Lymphocytic Leukemia; DLBCL, Diffuse Large B cell Lymphoma; EMT: Epithelial-Mesenchymal Transition; EGFR: Epidermal Growth Factor Receptor; GTPase: Guanosine Triphosphatase; IBD, Inflammatory Bowel Disease; IHC: Immunohistochemistry; MAIT, Mucosal-Associated Invariant T cells; NSCLC: Non-Small Cell Lung Cancer; PDAC: Pancreatic Ductal Adenocarcinoma; ROS: Reactive Oxygen Species; TILs: Tumor-Infiltrating Lymphocytes.

### Integrating protein and gene data to model PDAC progression under FOLFIRINOX

In the second model, which comprised gene expression profiling (GEP) and IPEP data, we employed BGLR modeling to address the challenge posed by a high-dimensional dataset where the number of variables greatly exceeded the number of samples (p >> n). Feature selection focused on identifying those with the highest impact on the models’ explained variability and ranking them using back-solving techniques. **Supplementary Table S2** presents the frequency of feature selection and their layer (gene or protein) following 50 iterations. Notably, no genes appeared in the top 60 ranks, suggesting that the IPEP data predominantly drove the predictive capacity of our prediction model. The incremental AUC analysis in this model revealed a risk of overfitting when selecting more than eight proteins, comparable to the first model (**Supplementary Figure S3**). The top eight ranked proteins included in this model were BANK1, CCL17, CCL20, FCRL3, MYO9B, PNLIPRP2, PRSS8, and TNFAIP8 (Table 2, **Supplementary Figure S4**) and were combined into a Multivariate Logistic Regression model to predict PDAC progression under FOLFIRINOX. The ROC performance analysis yielded an AUC [95 %CI] of 0.96 [0.91 – 1.00] in the training set and 0.81 [0.56 – 1.00] in the independent test set, both assessed separately from the cross-validation (Fig. 2A).

### A liquid signature of inflammatory proteins predicts PDAC progression under FOLFIRINOX

**Fig. 3:**
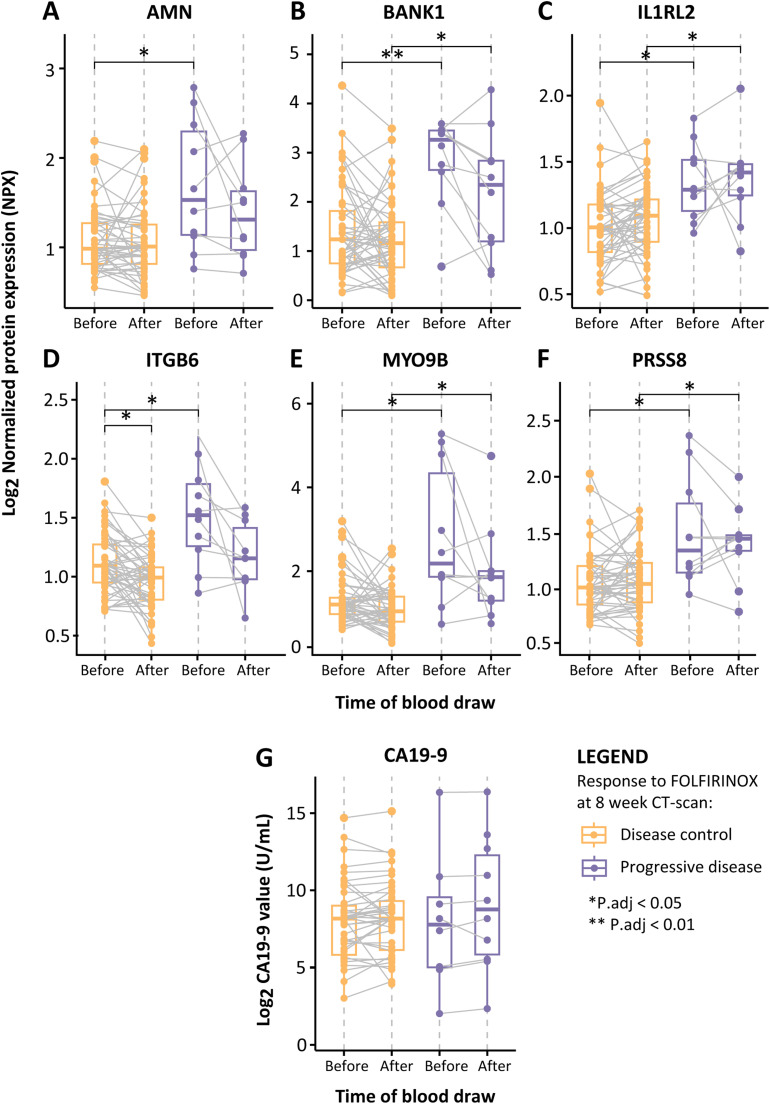
**Boxplots of the FFX-IPEP proteins and CA19-9 levels before and after the first FOLFIRINOX cycle. (A-F)** Boxplots displaying the levels of the six proteins included in the FFX-IPEP signature, stratified by treatment response (disease control and progressive disease) and the time of blood draw (x-axis). The y-axis displays log2 NPX values, with each dot representing the NPX value in an individual patient. **(G)** Boxplot displaying log10 CA19-9 values in U/mL (y-axis), stratified by treatment response (disease control and progressive disease) and the time of blood draw (x-axis). *P.adj < 0.05, **P.adj < 0.01. Abbreviations: AMN, Vitamin Transport Protein Amnionless; BANK1, B Cell Scaffold Protein with Ankyrin Repeats 1; CA19-9, Carbohydrate Antigen 19-9; IL1RL2, Interleukin 1 Receptor-Like 2; ITGB6, Integrin Beta-6; NPX, Normalized Protein Expression; MYO9B, Integrin Beta-6; PRSS8, Serine Protease 8.

A consensus model, defined as the FOLFIRINOX IPEP (FFX-IPEP) signature, was constructed as both variable selection methods consistently demonstrated models with similar predictive ability but slight differences in protein lists. The FFX-IPEP signature comprised proteins consistently selected in both models, appearing at least 10 times: AMN, BANK1, IL1RL2, ITGB6, MYO9B, and PRSS8 (Table 2). At baseline, all these proteins were significantly increased in progressive disease patients (P.adj < 0.05), and except for AMN and IL1RL2, this persisted after the first cycle of FOLFIRINOX (Fig. 3A-3F). Subsequently, these proteins were integrated into a Multivariate Logistic Regression model that adjusted for the covariates age, sex, and disease stage. Subsequently, regression coefficients (beta values) were assigned to the proteins to find the optimal combination of proteins (**Supplementary Table S3**). The following equation represents the FFX-IPEP signature:

FOLFIRINOXinflammatoryproteinexpressionprofiling(FFX−IPEP)signature=(1.14*AMN)+(0.62*BANK1)+(1.99*IL1RL2)+(1.35*ITGB6)+(0.50*MYO9B)+(1.21*PRSS8)

The calculated VIF values revealed no collinearity among the variables included in the model (**Supplementary Table S4**). The performance to predict PDAC progression under FOLFIRINOX was assessed by ROC analysis and yielded an AUC [95 % CI] of 0.94 [0.88 – 1.00] for the training set and 0.89 [0.74 – 1.00] for the independent test set, each assessed separately from the cross-validation (Fig. 2A). We also assessed the predictive capability of the absolute normalized CA19-9 tumor load marker levels for PDAC (Fig. 3G), which exhibited the lowest AUC [95 % CI] of 0.54 [0.38 – 0.79] in the independent test set. Upon combining our consensus model with the CA19-9 marker, the predictive capability decreased to an AUC [95 % CI] of 0.63 [0.45 – 0.90] (Fig. 2A). Importantly, there is less overlap in FFX-IPEP scores between disease control and progressive disease patients compared to CA19-9 scores. These findings suggest that our model outperformed the CA19-9 marker in predicting PDAC progression under FOLFIRINOX (Fig. 2B, 2C).

## Discussion

In this study, we examined the blood expression profiles of 348 proteins and 730 genes involved in immune or oncogenic processes from 58 PDAC patients before and after their first cycle of FOLFIRINOX. A liquid biomarker signature of multiple inflammatory proteins, the FFX-IPEP signature, was constructed with promising potential for early prediction of PDAC progression during FOLFIRINOX. This FFX-IPEP signature outperformed the conventional CA19-9 tumor load marker, and combining both did not yield enhanced predictive performances. Importantly, our predictive models accounted for disease stage, age, and sex, eliminating the need to consider disease stage for FOLFIRINOX response prediction. Furthermore, all patients underwent at least four FOLFIRINOX cycles, the time at which response was radiologically assessed, and none received alternative chemotherapies before initiating FOLFIRINOX.

Identifying patients who will or will not benefit from FOLFIRINOX is crucial as it is a treatment with superior efficacy among chemotherapy options for PDAC, albeit at the cost of high toxicity. Various liquid biomarkers for predicting chemosensitivity or resistance in PDAC have been explored over the years, including conventional tumor markers (CA19-9, CEA, SPAN-1), genetic markers (single nucleotide polymorphisms, circulating tumor DNA, long-non-coding RNAs), and immunological markers (NLR, SII, cytokines, or proteins, immune cells) [20]. Nevertheless, these markers have demonstrated restricted predictive accuracy, clinical utility, and limited specificity for FOLFIRINOX. Furthermore, anticipating a single marker to predict treatment outcomes for all diverse patient populations is unrealistic. Our earlier research highlighted the significance of a multigene model [19] and standalone protein biomarkers [22,23] in guiding FOLFIRINOX treatment decisions.

Building upon this, our present study aimed to improve the prediction of PDAC progression during FOLFIRINOX treatment by employing a multi-omics modeling approach. When comparing our first model using only IPEP data to our second model integrating GEP and IPEP data, proteins alone showed superior predictability. Noteworthy, these outcomes may vary depending on the subset of genes and proteins selected for analysis. Consequently, the third consensus model, the FFX-IPEP signature, exclusively utilized IPEP data due to its superior predictability. This biomarker signature achieved an AUC of 0.89 in an independent test set, effectively identifying patients transitioning from disease control to progression observed on CT scans conducted four weeks after initiating FOLFIRINOX. Compared to our previously proposed FFX-ΔGEP score [19], the improvement in predictive performance appears modest. Nonetheless, the current plasma-protein-based signature offers additional advantages over gene-based models, including cost-effective integration into clinical practice. Furthermore, not all the proteins in our current multiprotein model overlapped with previously identified individual proteins [23]. This may be due to the inclusion of patients with both GEP and IPEP in this study or the synergistic effect of protein combinations on their predictive capability. In this study, we deviated from our previous works by incorporating the time of blood draw as an interaction factor rather than calculating delta values. This approach offers multiple advantages, including preserving information from both time points, providing greater flexibility in modeling complex relationships between variables, and enhancing robustness against data variability and measurement errors.

The FFX-IPEP signature comprises six proteins: AMN, BANK1, IL1RL2, ITGB6, MYO9B, and PRSS8. At baseline, all these proteins were significantly upregulated in patients with progressive disease, which aligns with their oncogenic roles (Table 2). The Amnionless (AMN) protein has been linked to colorectal carcinogenesis and is associated with poor survival [32]. Interleukin 1 Receptor-Like 2 (IL1RL2) is a receptor for IL36, and this signaling axis plays a pro-tumorigenic role in colon cancer, contributing to poor survival [56,57]. The cell surface protein Integrin αvβ6 (ITGB6) promotes PDAC progression by influencing cellular behavior linked to worse survival [59], [60], [61]. Similarly, Myosin IXB (MYO9B) is implicated in the progression of lung and prostate cancer, enhancing cancer cell migration and reducing survival [63,64]. Furthermore, MYO9B knockdown in breast cancer cells suppressed their in vitro proliferation, migration, and invasiveness [65]. Notably, in our prior study focusing on standalone circulating biomarkers, ITGB6 and MYO9B demonstrated potential in early prediction of PDAC progression during FOLFIRINOX [23]. The serine protease enzyme (PRSS8) has exhibited anti-tumorigenic effects in bladder [73], breast [70], colorectal [71], and hepatocellular cancer [72]. Contrastingly, it has also been associated with paclitaxel and cisplatin chemoresistance in bladder [74] and ovarian cancer [75]. The sole exception among these proteins is the B Cell Scaffold Protein with Ankyrin Repeats 1 (BANK1), which predominantly displays anti-tumoral functions in B cell lymphoma [37]. However, considering its primary role in B cell signaling and the dual capacity role of B cells in PDAC development, the specific role of BANK1 remains undetermined [86,87].

Despite the robust scientific foundation of our prospective cohort study, ensuring a meticulous acquisition of sequential and accurate clinical data as well as rigorous application of advanced statistical methodologies, we acknowledge several limitations. First, the imbalance in patient distribution between disease control and progressive disease existed. This is partly due to our focus on early prediction, as, fortunately, few patients exhibit progressive radiological responses after only four cycles. This issue was addressed by creating balanced folds based on the response to FOLFIRINOX, enhancing the robustness of our performance assessments. Moreover, among the various models explored to address this imbalance most effectively, the current model consistently exhibited optimal performance with superior predictive capacity. Nevertheless, a validation study in an external cohort following REMARK guidelines [88] must establish an appropriate model cut-off value to facilitate clinical decision-making. Second, exploring how the models behaved in the distinct disease stage groups would have been interesting, but the limited sample size rendered this impossible. Instead, we addressed this limitation by adjusting all models for age, disease stage, and sex, thereby mitigating the influence of these factors on expression data. This approach holds clinical relevance, as the models now apply universally to the entire patient population rather than specific subsets. In future research, we intend to explore the model's behavior in the context of combination therapies with FOLFIRINOX and evaluate its specificity to FOLFIRINOX by testing it on patients undergoing different chemotherapy regimens. However, the current study was hindered by the restricted availability of patients receiving alternative chemotherapy regimens, given that FOLFIRINOX remains the preferred treatment. Third, the administration of G-CSF, which stimulates granulocyte production, might have influenced the patients' protein profiles [19]. However, G-CSF is standard practice in the Erasmus Medical Center to reduce the risk of neutropenia and is not believed to impact PDAC progression [24]. Lastly, while blood samples after only one cycle are optimal for early response prediction, longitudinal on-treatment samples would have enriched insight into the sustained viability and predictive capacity of our models for long-term prognosis.

## Conclusions

In summary, our six-protein FFX-IPEP signature is a liquid biomarker with a solid potential to predict PDAC progression early during FOLFIRINOX treatment. In our cohort, the FFX-IPEP signature predicted PDAC progression during FOLFIRINOX more accurately than changes in CA19-9. Future studies should expand to predict progression under different chemotherapies. This expansion could guide more effective personalized treatment selection while at the same time avoiding ineffective but toxic treatment.

## Data availability

The datasets used and analyzed during the current study are available, with permission of the Erasmus Medical Center Rotterdam, from the corresponding author on reasonable request.

## Ethics approval and consent to participate

The participating patients in this study originated from the phase III PREOPANC-2 RCT (Dutch trial register NL7094) and the iKnowIT prospective cohort study (Dutch trial register NL7522). Both studies adhered to the Declaration of Helsinki and were approved by the Ethics Committees of the Erasmus MC, with the PREOPANC-2 trial approved in June 2018 (MEC-2018-004) and the iKnowIT study approved in February 2019 (MEC-2018-087). Written informed consent was obtained from all patients.

## Funding

This work was supported by the Survival with Pancreatic Cancer Foundation (www.supportcasper.nl) [grant number OVIT17-06].

## CRediT authorship contribution statement

**Casper W.F. van Eijck:** Conceptualization, Formal analysis, Investigation, Methodology, Software, Validation, Visualization, Writing – original draft, Writing – review & editing. **Sergio Sabroso-Lasa:** Conceptualization, Formal analysis, Investigation, Methodology, Software, Validation, Visualization, Writing – original draft, Writing – review & editing. **Gaby J. Strijk:** Investigation, Resources, Writing – review & editing. **Dana A.M. Mustafa:** Conceptualization, Resources, Writing – review & editing. **Amine Fellah:** Investigation, Resources, Writing – review & editing. **Bas Groot Koerkamp:** Conceptualization, Project administration, Writing – review & editing. **Núria Malats:** Conceptualization, Methodology, Supervision, Writing – original draft, Writing – review & editing. **Casper H.J. van Eijck:** Conceptualization, Funding acquisition, Methodology, Project administration, Supervision, Writing – original draft, Writing – review & editing.

## Declaration of competing interest

The authors declare that they have no known competing financial interests or personal relationships that could have appeared to influence the work reported in this paper.

## Appendix Supplementary materials

Image, application 1

Image, application 2

Image, application 3

Image, application 4

Image, application 5

Image, application 6
