# Supplementary material for: A liquid biomarker signature of inflammatory proteins accurately predicts early pancreatic cancer progression during FOLFIRINOX chemotherapy
Source: Neoplasia. 2024 Feb 9;49:100975. doi: 10.1016/j.neo.2024.100975 (PMC10873733; doi:10.1016/j.neo.2024.100975)

Supplementary Figure S1

AUC according to the number of features in the first model (exclusively using protein expression data)

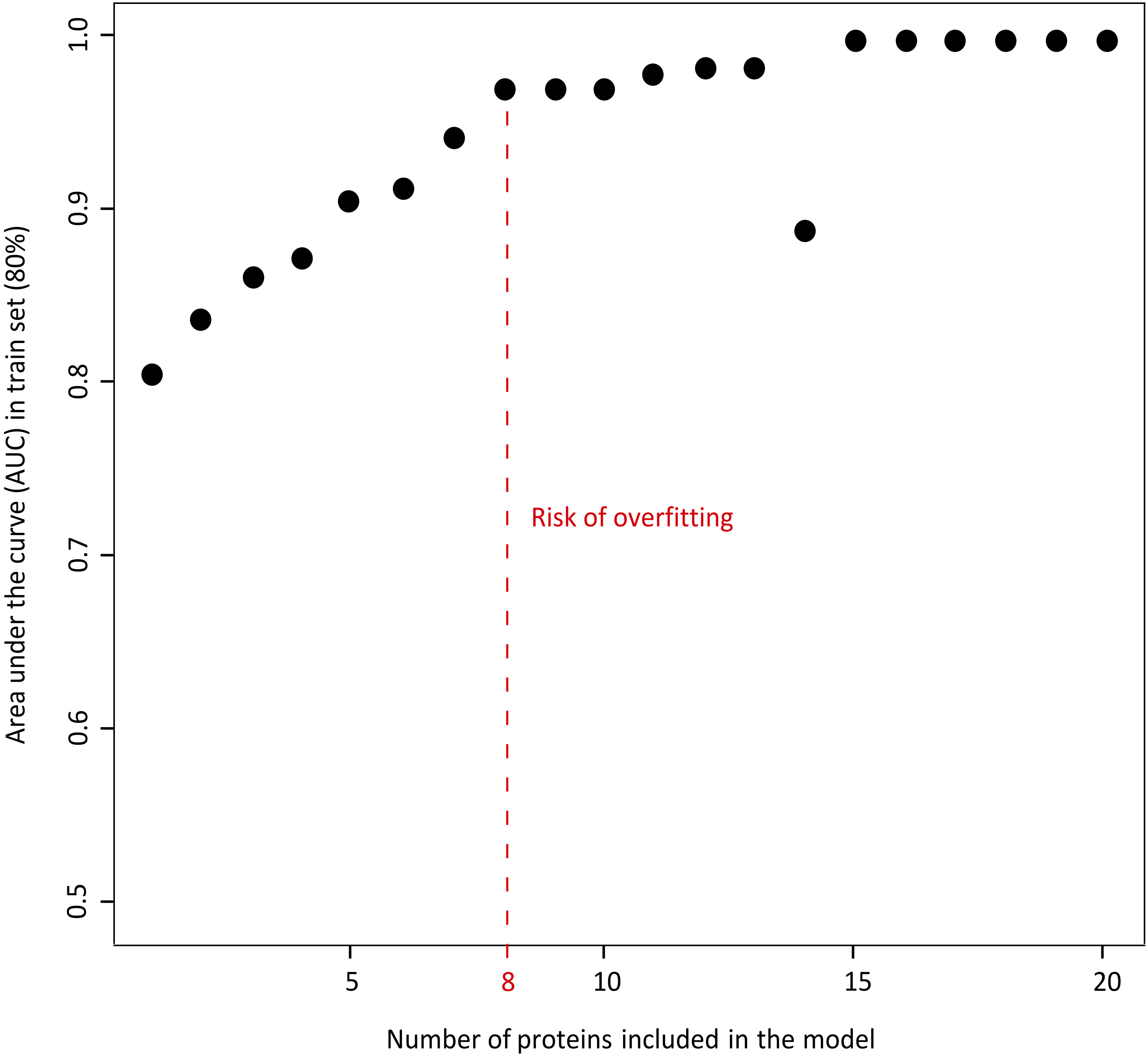

Supplement: Supplementary file 1 [file mmc1.pdf]
