# Supplementary material for: A liquid biomarker signature of inflammatory proteins accurately predicts early pancreatic cancer progression during FOLFIRINOX chemotherapy
Source: Neoplasia. 2024 Feb 9;49:100975. doi: 10.1016/j.neo.2024.100975 (PMC10873733; doi:10.1016/j.neo.2024.100975)

Supplementary Figure S3

AUC according to the number of features in the second model (using protein & gene expression data)

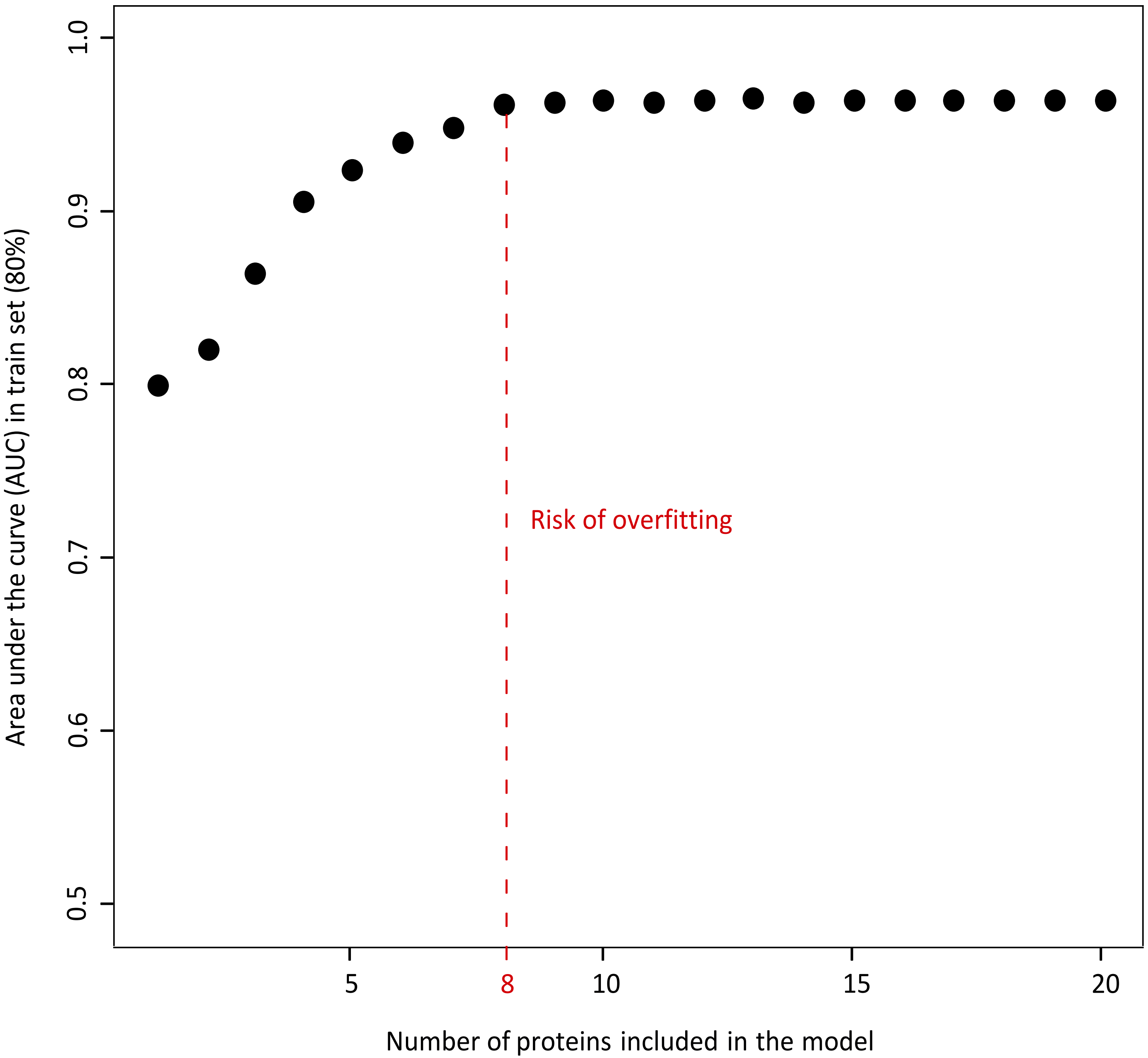

Supplement: Supplementary file 3 [file mmc3.pdf]
