# Supplementary material for: A liquid biomarker signature of inflammatory proteins accurately predicts early pancreatic cancer progression during FOLFIRINOX chemotherapy
Source: Neoplasia. 2024 Feb 9;49:100975. doi: 10.1016/j.neo.2024.100975 (PMC10873733; doi:10.1016/j.neo.2024.100975)

Supplementary Figure S4

Boxplots displaying the expression levels of features included in the second model (integrating gene and protein expression data)

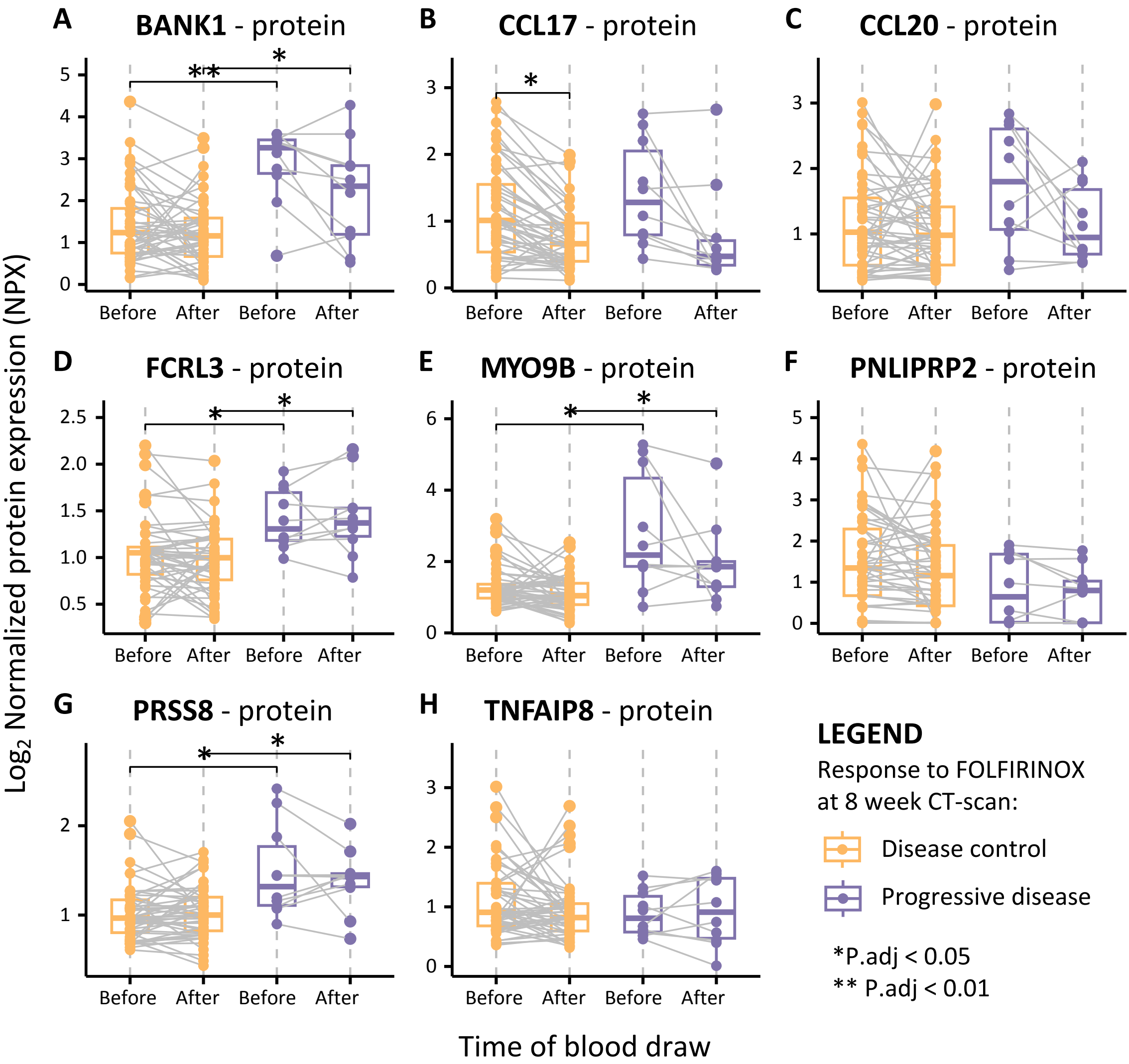

Supplement: Supplementary file 4 [file mmc4.pdf]
