# Supplementary material for: A liquid biomarker signature of inflammatory proteins accurately predicts early pancreatic cancer progression during FOLFIRINOX chemotherapy
Source: Neoplasia. 2024 Feb 9;49:100975. doi: 10.1016/j.neo.2024.100975 (PMC10873733; doi:10.1016/j.neo.2024.100975)
Supplement: Supplementary file 5 [file mmc5.docx]

# Supplementary Materials

## Targeted multiplex gene expression profiling (GEP) using the NanoString nCounter platform

Two Tempus tubes per patient (one for each time point) were thawed and subjected to gene expression profiling using the nCounter FLEX system of NanoString Technologies (Seattle, WA, USA). The PanCancer Immune profiling panel^1^ was used, which quantifies the expression of 40 housekeeping genes and 730 genes, as described previously.^2^ Total RNA was extracted from blood using the Tempus Spin RNA Isolation Kit of Thermo Fisher Scientific (Waltham, MA, USA) following the manufacturer’s instructions. RNA quality was assessed using the Agilent 2100 BioAnalyzer (Santa Clara, CA, USA), and samples with RNA concentrations below 35 mg/mL were excluded. To correct for RNA degradation, corrected RNA concentrations were calculated based on the percentage of fragments of 300-4000 nucleotides. A maximum of 7 μL of 200 ng RNA per sample was used for hybridization, which was performed at 65°C for 17 hours using the SimpliAmp Thermal Cycler from Applied Biosystems (Applied Biosystems). Gene expression was quantified by scanning 490 Fields of View (FOV). Normalization, standardization, and quality control were performed using the nSolver software (version 4.0) and the Advanced Analysis module (version 2.0).^3^ Gene expression data were only included if all positive and negative control genes fell within the expected values and if binding density values ranged between 0.5 and 3.0. Raw gene counts were then normalized using the geNorm algorithm,^4^ based on the stable housekeeping genes. All normalized data were log2 transformed, and genes were only included when they were higher than the limit of detection of 4.4 log2 in more than 80% of samples.

## Plasma inflammatory protein expression profiling (IPEP) using the Olink Proteomics platform

Two Vacutainer serum separator tubes per patient (one for each time point) were thawed and subjected to proteomic profiling using the Olink Explore 348 Inflammation panel. This panel quantified the plasma concentrations of 348 proteins according to the manufacturer’s protocol as described previously.^5^ Olink Proteomics (Uppsala, Sweden) uses the Proximity Extension Assay (PEA) technology^6^ to detect nucleotide-labeled antibody probe pairs to individual proteins in real-time PCR^7, 8^ Following normalization, standardization, and quality assessment using the Olink Proteomics pipeline,^9^ final protein quantities were presented as normalized protein expression (NPX) values with a pseudocount of +1 to enable log2 transformation. Patient samples with a deviation of less than 0.3*NPX from the median of their internal control passed the assessment. Downstream statistical analysis was only performed on proteins detected in more than 75% of patient samples and with less than 25% of values below the limit of detection (LOD). When values were below LOD, actual NPX values were used to impute best-guess values. If necessary, principal component analyses (PCAs) were used to identify and exclude possible sample outliers.

# References

1. Cesano A. nCounter((R)) PanCancer Immune Profiling Panel (NanoString Technologies, Inc., Seattle, WA). *J Immunother Cancer*. 2015;3:42. doi:10.1186/s40425-015-0088-7

2. van Eijck CWF, de Koning W, van der Sijde F, et al. A multigene circulating biomarker to predict the lack of FOLFIRINOX response after a single cycle in patients with pancreatic ductal adenocarcinoma. *Eur J Cancer*. Mar 2023;181:119-134. doi:10.1016/j.ejca.2022.12.024

3. Geiss GK, Bumgarner RE, Birditt B, et al. Direct multiplexed measurement of gene expression with color-coded probe pairs. *Nat Biotechnol*. Mar 2008;26(3):317-25. doi:10.1038/nbt1385

4. Vandesompele J, De Preter K, Pattyn F, et al. Accurate normalization of real-time quantitative RT-PCR data by geometric averaging of multiple internal control genes. *Genome Biol*. Jun 18 2002;3(7):RESEARCH0034. doi:10.1186/gb-2002-3-7-research0034

5. van Eijck CWF, Strijk G, Vietsch EE, et al. FOLFIRINOX chemotherapy modulates the peripheral immune landscape in pancreatic cancer: Implications for combination therapies and early response prediction. *Eur J Cancer*. Nov 17 2023;196:113440. doi:10.1016/j.ejca.2023.113440

6. Assarsson E, Lundberg M, Holmquist G, et al. Homogenous 96-plex PEA immunoassay exhibiting high sensitivity, specificity, and excellent scalability. *PLoS One*. 2014;9(4):e95192. doi:10.1371/journal.pone.0095192

7. Bettoli V, Join-Lambert O, Nassif A. Antibiotic Treatment of Hidradenitis Suppurativa. *Dermatol Clin*. Jan 2016;34(1):81-9. doi:10.1016/j.det.2015.08.013

8. Whelan CD, Mattsson N, Nagle MW, et al. Multiplex proteomics identifies novel CSF and plasma biomarkers of early Alzheimer's disease. *Acta Neuropathol Commun*. Nov 6 2019;7(1):169. doi:10.1186/s40478-019-0795-2

9. Proteomics O. White paper - Data normalization and standardization. Upsala, Sweden2021. p. 1-8.
